# Supplementary material for: Bi-Directional, Day-to-Day Associations between Objectively-Measured Physical Activity, Sedentary Behavior, and Sleep among Office Workers
Source: Int J Environ Res Public Health. 2021 Jul 28;18(15):7999. doi: 10.3390/ijerph18157999 (PMC8345408; doi:10.3390/ijerph18157999)
Supplement: Supplementary file 1 [file ijerph-18-07999-s001.zip › ijerph-1292375-supplementary.pdf]

International Journal of
Environmental Research and Public Health

MDPI

Supplementary Materials

Supplementary Statistical Analysis

In order to examine bi-directional, day-to-day, within-person associations between physical activity, sedentary behavior, and sleep, multilevel linear mixed modeling (MLM) for repeated measures were employed [32], in a similar manner as described in Dzierzewski et al. [33]. All the MLMs were estimated using restricted maximum likelihood with an unstructured variance-covariance structure. All dependent and main independent variables were standardized into z-scores prior to estimation of the MLM to facilitate interpretation of parameters. MVPA and SE were skewed and therefore log transformed before standardization. Two sets of MLMs were run to study the intra-individual differences. In all models MVPA and sedentary behavior were mutually controlled for.

The first set of MLMs was parametrized to test whether daily daytime MVPA and sedentary behavior (independent variables) were associated that night's TST and SE (dependent variables). MVPA or sedentary behavior were introduced in the MLMs as person-centered daily variations as both fixed and random effects of these behaviors (i.e. within-person random effects computed as the variation of time spent in either MVPA or sedentary behavior around each person's weekly averages [e.g., person-centered MVPA – average MVPA]). In the models, average levels of the independent variables were also controlled for and models included random effects and an error term. Here are the equations for the first set of MLMs:

MVPA → TST

$TST_{it} = \beta_0 + \beta_1$ (person-centered $MVPA_i$) + β_2 (average $MVPA_i$) + β_3 (average $Sedentary_i$) + β_4 (person-centered $Sedentary_i$) + r_{1i} (person-centered MVPA) + r_{0i} + e_{it}

Sedentary →TST

$TST_{it} = \beta_0 + \beta_1$ (person-centered $MVPA_i$) + β_2 (average $MVPA_i$) + β_3 (average $Sedentary_i$) + β_4 (person-centered $Sedentary_i$) + r_{1i} (person-centered Sedentary) + r_{0i} + e_{it}

MVPA →SE

$SE_{it} = \beta_0 + \beta_1$ (person-centered $MVPA_i$) + β_2 (average $MVPA_i$) + β_3 (average $Sedentary_i$) + β_4 (person-centered $Sedentary_i$) + r_{1i} (person-centered MVPA) + r_{0i} + e_{it}

Sedentary →SE

$SE_{it} = \beta_0 + \beta_1$ (person-centered $MVPA_i$) + β_2 (average $MVPA_i$) + β_3 (average $Sedentary_i$) + β_4 (person-centered $Sedentary_i$) + r_{1i} (person-centered Sedentary) + r_{0i} + e_{it}

The second set of MLMs was to test whether sleep parameters (TST and SE) were associated with MVPA or sedentary behavior the following day by shifting the dataset to match sleep with the next day's physical activity/sedentary behavior in the same row. MVPA and sedentary behavior were the dependent variables and sleep parameters from the previous night were independent variables, included as person-centered fixed and random effects. Here are the equations for the second set of MLMs:

TST → MVPA

$MVPA_{it} = \beta_0 + \beta_1$ (person-centered TST_i) + β_2 (average TST_i) + β_3 (average $Sedentary_i$) + β_4 (person-centered $Sedentary_i$) + r_{1i} (person-centered TST) + $r_{0i} + e_{it}$

TST → Sedentary

$Sedentary_{it} = \beta_0 + \beta_1$ (person-centered TST_i) + β_2 (average TST_i) + β_3 (average $MVPA_i$) + β_4 (person-centered $MVPA_i$) + r_{1i} (person-centered TST) + $r_{0i} + e_{it}$

SE → MVPA

$MVPA_{it} = \beta_0 + \beta_1$ (person-centered SE_i) + β_2 (average SE_i) + β_3 (average $Sedentary_i$) + β_4 (person-centered $Sedentary_i$) + r_{1i} (person-centered SE) + $r_{0i} + e_{it}$

SE → Sedentary

$Sedentary_{it} = \beta_0 + \beta_1$ (person-centered SE_i) + β_2 (average SE_i) + β_3 (average $MVPA_i$) + β_4 (person-centered $MVPA_i$) + r_{1i} (person-centered SE) + $r_{0i} + e_{it}$

In model 1 MVPA and sedentary behavior were mutually controlled for. In model 2 subject-level covariates (i.e. age, sex, cognitive performance, fitness) were additionally controlled for. Interactions were tested between the main independent variables and potential modifiers (i.e. age, sex, fitness, cognitive performance, and weekend/weekday). Weekend/weekday was used in the models as a binary dummy variable. Subsequent stratified MLMs were performed for statistically significant interactions ($p < 0.1$). Statistical significance was set at a p-value <0.05. Statistical software Stata version 15 (StataCorp, College Station, TX, USA) was used for all analyses.

Supplementary Tables

Table S1. Associations between physical activity, sedentary behavior, and sleep, taking stress and education into account as potential confounders.

	Standardized Beta-Coefficient (95% confidence interval)			
	**Model 1**	**Model 2**	**Model 1**	**Model 2**
	Total Sleep Time		Sleep efficiency	
Sedentary behavior	−0.14 (−0.18, −0.10)**	−0.14 (−0.18, −0.10)**	0.04 (−0.002, 0.07)	0.04 (−0.001,0.07)
MVPA	−0.03 (−0.08, 0.02)	−0.03 (−0.08, 0.01)	−0.001 (−0.04, 0.03)	−0.001 (−0.04, 0.03)
	Sedentary behavior		MVPA	
Total Sleep Time	−0.14 (−0.18, −0.10)**	−0.14 (−0.18, −0.10)**	−0.07 (−0.11, −0.03)**	−0.07 (−0.11, −0.03)**
Sleep Efficiency	0.04 (0.01, 0.07)*	0.04 (0.01, 0.07)*	0.03 (−0.003, 0.07)	0.03 (−0.004, 0.07)

Model 1: controlled for MVPA/ sedentary behavior, age, sex, fitness, cognitive performance, and stress; Model 2: controlled for Model 1 and education. Missing 22 in stress variable. MVPA: moderate-to-vigorous physical activity; TST: total sleep time; SE: sleep efficiency. p-value * ≤0.05, ** ≤0.001. Variables were z-score transformed prior to model parameterization. Stress was assessed as a single-item measure on a 5-point Likert scale[31], where the participant was asked "Do you feel this kind of stress these days? Stress defined as a situation in which a person feels tense, restless, nervous or anxious, or is unable to sleep at night because their mind is troubled all the time".

Table S2. Associations between physical activity, sedentary behavior, and sleep using logistic mixed-effects models.

	**Standardized Beta-Coefficient (95% confidence interval)**	
	**Model 1**	**Model 2**
	Sleep efficiency	
Sedentary behavior	0.04 (−0.07, 0.15)	0.06 (−0.06, 0.18)
Moderate-to-vigorous physical activity	−0.06 (−0.18, 0.05)	−0.05 (−0.17, 0.07)
	Moderate-to-vigorous physical activity	
Total Sleep Time	−0.01 (−0.20, 0.01)	−0.09 (−0.20, 0.01)
Sleep Efficiency	0.10 (−0.02, 0.22)	0.08 (−0.04, 0.21)

Model 1: control for moderate-to-vigorous physical activity or sedentary behavior; Model 2: control for Model 1 and age, sex, fitness, and cognitive performance. Variables were z-score transformed prior to model parameterization.

Table S3. The association between daytime moderate-to-vigorous physical activity and sleep efficiency that night, stratified by sex, using logistic mixed-effects models.

	**Standardized Beta-Coefficient for sleep efficiency (95% confidence interval)**			
	**Women**		**Men**	
	Model 1	Model 2	Model 1	Model 2
Moderate-to-vigorous physical activity	0.02 (−0.12, 0.16)	0.03 (−0.11, 0.18)	−0.207 (−0.40, −0.01)*	−0.19 (−0.39, 0.01)

Model 1: control for moderate-to-vigorous physical activity or sedentary behavior; Model 2: control for Model 1 and age, sex, fitness, and cognitive performance. *p*-value * ≤0.05. Variables were z-score transformed prior to model parameterization.
